# Prolactin Acts on Myeloid Progenitors to Modulate SMAD7 Expression and Enhance Hematopoietic Stem Cell Differentiation into the NK Cell Lineage

**DOI:** 10.1038/s41598-020-63346-4

**Published:** 2020-04-14

**Authors:** Dejene M. Tufa, Tyler Shank, Ashley M. Yingst, George Devon Trahan, Seonhui Shim, Jessica Lake, Renee Woods, Kenneth Jones, Michael R. Verneris

**Affiliations:** University of Colorado and Children’s Hospital of Colorado, Department of Pediatrics, Center for Cancer and Blood Disorders. Research Complex 1, North Tower, 12800 E. 19th Ave., Mail Stop 8302, Room P18-4108, Aurora, CO 80045 USA

**Keywords:** Transforming growth factor beta, Lymphopoiesis, NK cells

## Abstract

Numerous cell types modulate hematopoiesis through soluble and membrane bound molecules. Whether developing hematopoietic progenitors of a particular lineage modulate the differentiation of other hematopoietic lineages is largely unknown. Here we aimed to investigate the influence of myeloid progenitors on CD34^+^ cell differentiation into CD56^+^ innate lymphocytes. Sorted CD34^+^ cells cultured in the presence of stem cell factor (SCF) and FMS-like tyrosine kinase 3 ligand (FLT3L) give rise to numerous cell types, including progenitors that expressed the prolactin receptor (PRLR). These CD34^+^PRLR^+^ myeloid-lineage progenitors were derived from granulocyte monocyte precursors (GMPs) and could develop into granulocytes in the presence of granulocyte-macrophage colony-stimulating factor (GM-CSF) *in vitro*. Moreover, CD34^+^PRLR^+^ myeloid progenitors lacked lymphoid developmental potential, but when stimulated with prolactin (PRL) they increased the differentiation of other CD34^+^ cell populations into the NK lineage in a non-contact dependent manner. Both mRNA and protein analyses show that PRL increased mothers against decapentaplegic homolog 7 (SMAD7) in CD34^+^PRLR^+^ myeloid cells, which reduced the production of transforming growth factor beta 1 (TGF-β1), a cytokine known to inhibit CD56^+^ cell development. Thus, we uncover an axis whereby CD34^+^PRLR^+^ GMPs inhibit CD56^+^ lineage development through TGF-β1 production and PRL stimulation leads to SMAD7 activation, repression of TGF-β1, resulting in CD56^+^ cell development.

## Introduction

Hematopoietic differentiation is specified by a multitude of soluble and membrane-bound molecules produced both within and outside of the hematopoietic system that influence cell fate decisions^1–6^. In line with this, numerous cytokines including interleukin (IL)-1, IL-2, IL-7, IL-12, IL-15, IL-18, IL-21, IL-23, IL-25 and IL-33 have been shown to modulate the development of NK cells and other innate lymphoid cells (ILCs)^7–14^. Moreover, NK cells and other ILCs require different transcription factors such as Tbet, RORc and Gata3 for their development^7,15,16^. Multi-lymphoid progenitor (MLP) differentiate into the common ILC progenitor and this cell then gives rise to NK cells and all helper ILCs (i.e., ILC1, 2 and 3)^12–14,17^. NK cells and helper ILCs are distinguished by specific transcription factor expression, cytokine production and function^9,13,14,18,19^. We have used *in vitro* differentiation to study these processes and over the last decade have found that both NK cells and helper ILCs (particularly, ILC1s and ILC3s) develop in this system and similarly express CD56^16,20–23^. Therefore, throughout this manuscript we use the term CD56^+^ lymphocytes to describe all CD56 expressing cells.

Prolactin (PRL) is a neuroendocrine hormone best known for its role in lactation. However, PRL also regulates hematopoietic cell development and homeostasis^24–28^. Specifically, PRL enhances the development of myeloid and erythroid progenitors from CD34^+^ cells^24,26^. PRL also drives the maturation and activation of T cells, B cells, NK cells, neutrophils, macrophages and dendritic cells^27–33^. This hormone is released mainly by the anterior pituitary gland, although immune cells, such as myeloid cells, are non-endocrine sources of PRL^27,28,34,35^. PRL signals through the PRL receptor (PRLR), which is a member of the cytokine receptor superfamily^36–40^ because of its use of kinases and signal transduction activators of transcription (STATs)^36,38,41^. Apart from mammary gland tissue, decidua and uterus all of which abundantly express PRLR, immune cells also express this receptor^27,34,39,42,43^. Moreover, myeloid cells can co-express both PRL and its receptor (PRLR), indicating the existence of both autocrine and paracrine actions of this molecule within the hematopoietic system^26,27,34,44^.

The expression of PRLR in a subset of human CD34^+^ hematopoietic stem cells (HSCs) has previously been described and suggests a role for PRL during hematopoiesis^24–26,28^. In line with this, PRL directly promotes hematopoietic cell differentiation, accelerating immune reconstitution after bone marrow transplant (BMT)^24,28^. Studies also suggest the indirect involvement of PRL during lymphoid development, but the details remain unclear^28^. In this study, we report that stem cell factor (SCF) and FMS-like tyrosine kinase 3 ligand (FLT3L) induce the PRLR on CD34^+^ myeloid progenitors. We show that PRL acts on the CD34^+^PRLR^+^ myeloid progenitors resulting in the activation of pro-inflammatory factors such as IL-15 that support CD56^+^ lymphoid lineage development^45–47^. Mechanistically, we demonstrate that PRL increased mothers against decapentaplegic homolog 7 (SMAD7) which inhibits transforming growth factor beta (TGF-β) signaling by binding to and cleaving TGF-β receptor^48,49^. Moreover, the reduction in TGF-β1 following PRL stimulation is likely consistent with prior work showing SMAD7-induced negative-feedback regulation of TGF-β^48–50^. TGF-β inhibits NK cell development and function through inhibition of various metabolic pathways, including oxidative phosphorylation, glycolytic pathways, and respiratory pathways^50–53^. Thus, these studies show that PRL-induced SMAD7 facilitates CD56^+^ lymphocyte development through TGF-β repression.

## Results

### SCF and FLT3L Drive the Differentiation of HSCs into PRLR^+^CD34^+^ Myeloid Progenitors

**Figure 1:**
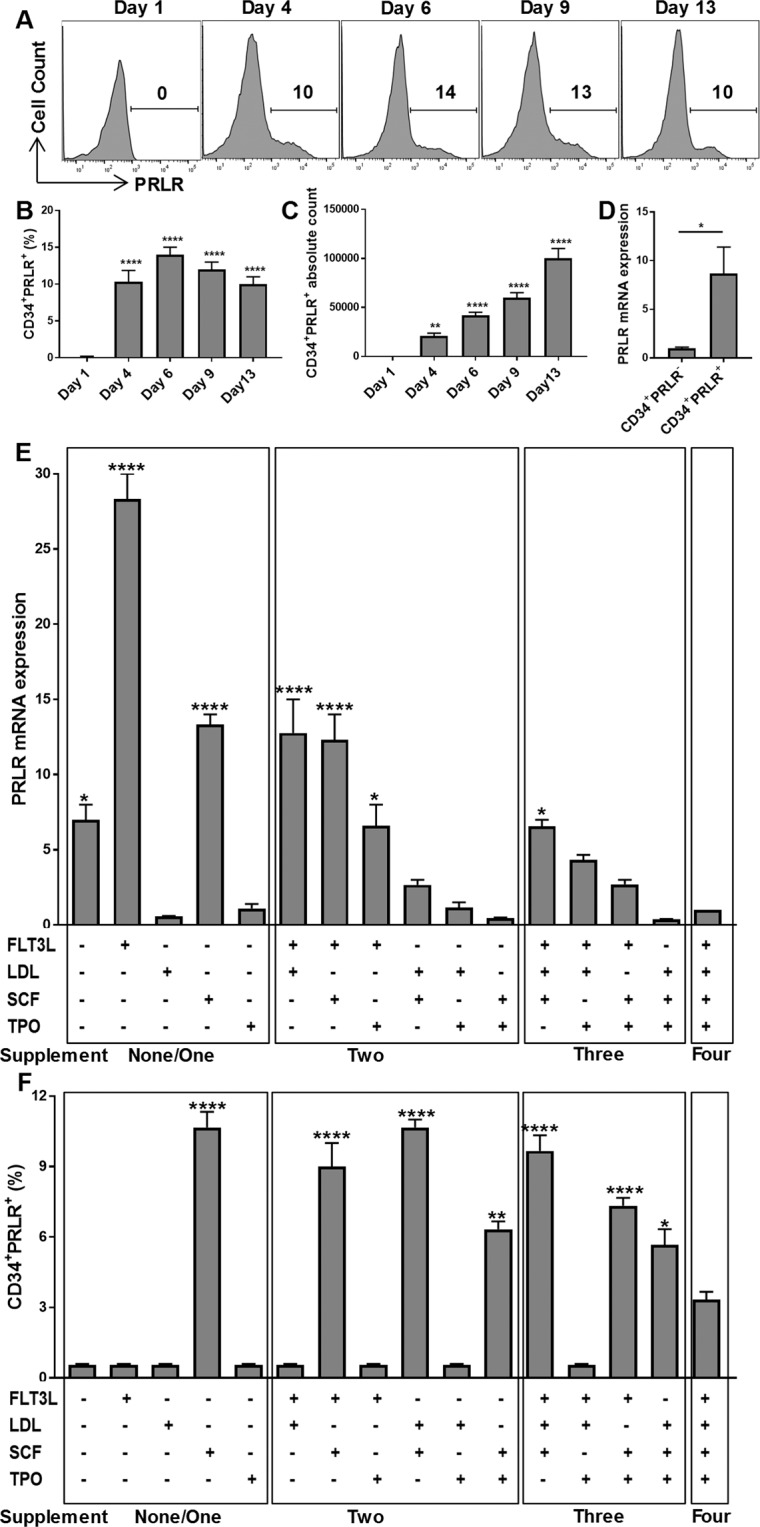
CD34^+^PRLR^+^ progenitors are present in cultures that favor CD56^+^ ILC differentiation. UCB-derived CD34^+^ HSCs were expanded for up to 13 days and the expression of PRLR was analyzed using qPCR or flow cytometry. (**A**) Expression of PRLR in differentiating HSCs at various time points. Representative histograms and values show the percentage of CD34^+^PRLR^+^ cells as assessed by FACS (n = 4). (**B,C**) The percentage (**B**) and absolute count (**C**) of CD34^+^PRLR^+^ progenitors in cultures at various time points is shown in bar graph (n = 4/group). (**D**) The quantitative expression of PRLR mRNA in CD34^+^PRLR^+^ cells is shown relative to its expression in CD34^+^PRLR^−^ cells after normalizing to GAPDH expression (n = 4/group). (**E,F**) CD34^+^ HSCs were expanded for 4 days using a combination of 3, 2 or one of the following: FLT3L, LDL, SCF and TPO, (**E**) The expression of PRLR mRNA in HSCs treated using a combination of 3, 2 or single supplements are shown, relative to the expression in HSCs that are treated with all 4 supplements after normalizing to GAPDH expression (n = 3/group), (**F**) HSCs were stained for the surface PRLR and the percentage of CD34^+^PRLR^+^ progenitors are shown in the bar graph (n = 3/group). (**B–F**) Data are shown as means ± SD, One-way ANOVA (**B,C,E and F**) or paired t-tests (**D**) and significance is depicted (E and F, only significant increases compared to HSCs that are treated with all 4 supplements is depicted) (* = p < 0.05; ** = p < 0.001; **** = p < 0.0001).

While studying *in vitro* differentiation of CD56^+^ lymphocytes from CD34^+^ progenitors, we noticed a minor population of non-ILC lineage cells that differentiated early in the cultures and were CD11a^low^ and negative for ILC markers including CD56, CD94, CD336, CD117 and CD294^16^. We sought to both characterize these cells and to determine whether they promoted or suppressed CD56^+^ lymphocyte development. Interestingly, these CD11a^low^ non-ILC cells expressed the PRLR (Supplementary Fig. 1). Freshly isolated cord blood CD34^+^ HSCs lacked the PRLR (Fig. 1A,B, Supplementary Fig. 2A), but ~15% of CD34^+^-derived cells acquire PRLR after a few days in media containing cytokines previously shown to expand HSCs (SCF, thrombopoietin (TPO), low-density lipoprotein (LDL) and FLT3L)^54^. Similarly, freshly isolated bone marrow and peripheral blood CD34^+^ HSCs lacked PRLR expression but acquired PRLR after four days of culture in media containing SCF, TPO, LDL and FLT3L (Supplementary 2B). The proportion of PRLR expressing progenitors was stable during the first two weeks of culture (Fig. 1A,B), while the absolute number significantly increased over time (Fig. 1C). Accordingly, these PRLR expressing progenitors upregulated PRLR mRNA (Fig. 1D). To understand the factors that drive PRLR expression, CD34^+^ cells were cultured in various cytokine combinations and PRLR mRNA and surface protein expression was tested. As shown in Fig. 1E, FLT3L significantly enhanced PRLR mRNA expression, while SCF (either alone or in combination) significantly increased surface PRLR expression (Fig. 1F).

### PRLR-expressing Progenitors Are Derived from GMPs and Differentiate into Mature Granulocytes under the Influence of GM-CSF

**Figure 2:**
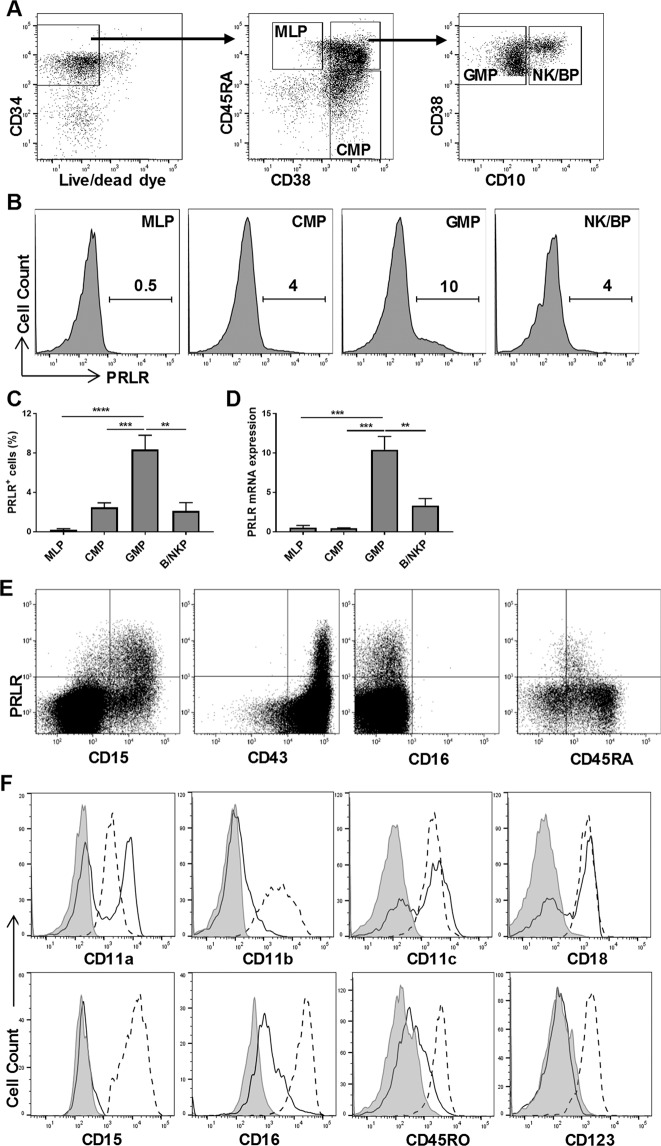
CD34^+^PRLR^+^ progenitors are derived from GMPs. Freshly isolated UCB-derived CD34^+^ HSCs were FACS sorted into MLP, CMP, GMP and NK/BP and expanded for four days using FLT3L, LDL, SCF and TPO cytokines. CD34^+^PRLR^+^ cells were sorted at day 4 and differentiated for up to 14 days (**A**) Gating strategy, freshly isolated CD34^+^ HSCs were sorted by FACS into MLPs, CMPs, GMPs and NK/BPs. (**B**) Expression of PRLR in various CD34^+^ HSC progenitors at day 4. Representative histograms and values represent the percentage of PRLR^+^ cells (n = 5). (**C**) Various HSC progenitors were stained for the surface PRLR at day 4 and the percentage of PRLR^+^ progenitors is shown in bar graph (n = 5/group). (**D**) The quantitative expression of PRLR mRNA in CMPs, GMPs and NK/BPs at day 4 are shown relative to its expression in MLPs after normalizing to the expression of GAPDH (n = 5/group). (**E**) Surface CD15, CD16, CD43 and CD45RA expression by CD34^+^PRLR^+^ cells at day 4 using flow cytometry. Representative dot plots (n = 5). (**F**) Surface CD11a, CD11b, CD11c, CD15, CD16, CD18, CD123 and CD45RO expression of day 14 differentiating CD34^+^PRLR^+^ cells (dashed-line), compared to day 14 differentiating CD34^+^PRLR^−^ cells (solid line) and the shaded histogram (isotype control) using flow cytometry. Representative histograms (n = 5). (**C,D**) Data are shown as means ± SD, One-way ANOVA and significance is shown (**=p < 0.001; ****=p < 0.0001). MLPs (multi lymphoid progenitors), CMPs (common myeloid progenitors), GMPs (granulocyte-monocyte progenitors) and NK/BPs (natural killer/B-cell progenitors).

**Figure 3:**
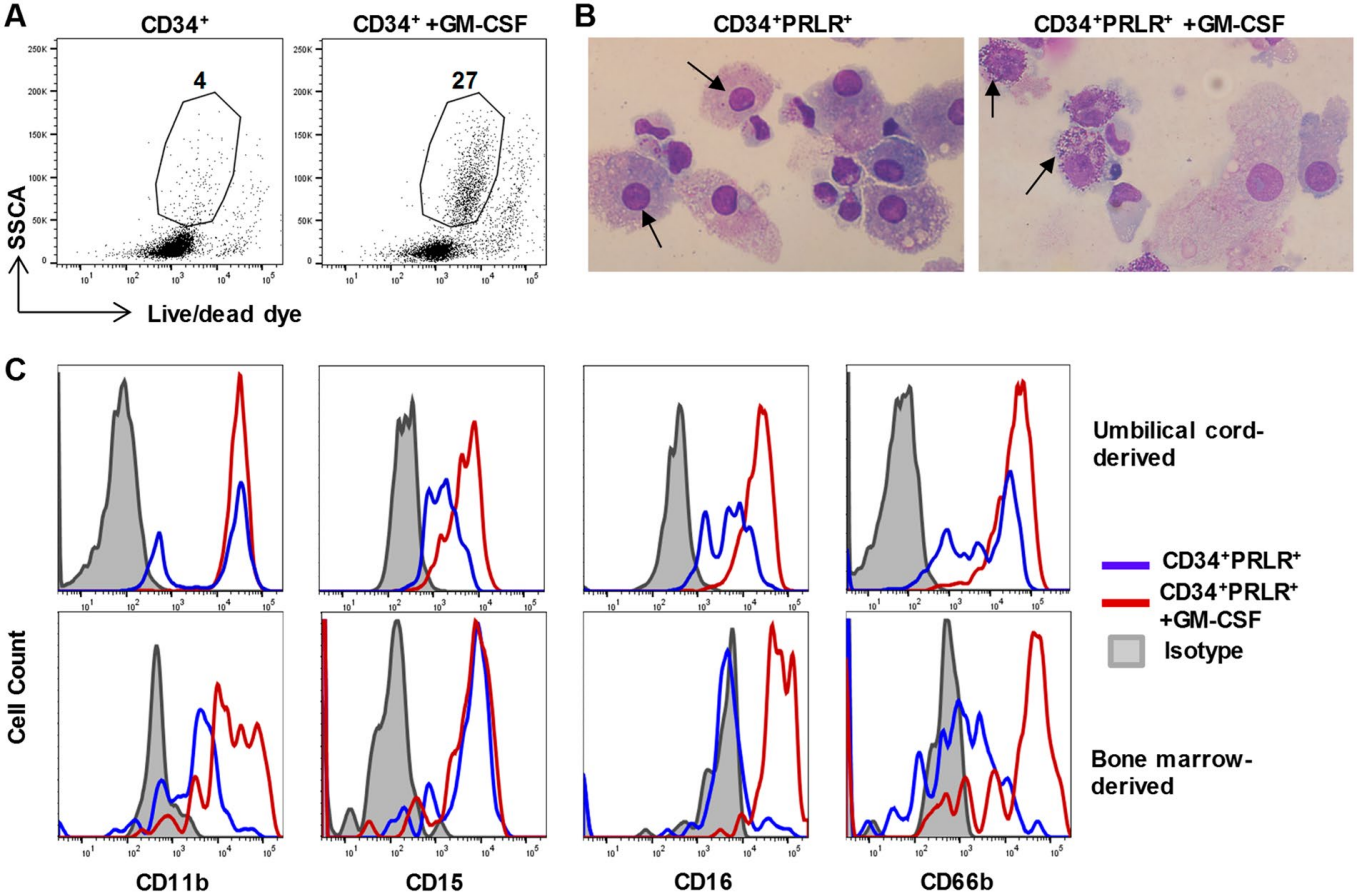
CD34^+^PRLR^+^ progenitors differentiate into mature granulocytes under the influence of GM-CSF. UCB-derived CD34^+^ HSCs were expanded for four days using FLT3L, LDL, SCF and TPO and differentiated for 21 days. Following this, cells were cultured for an additional 7 days of maturation in the presence or absence of GM-CSF. (**A**) Flow cytometry and dot plot (SSC vs live/dead) showing the granularity of live cells in GM-CSF treated vs untreated culture. Representative dot plots and values represent the percentage (n = 3). (**B**) Histologic analysis was performed on cells that underwent cytospin, methanol fixation and Wright’s staining. The images (arrow) of differentiating cells show immature (left) vs mature (right) granulocytes in the absence or presence of GM-CSF, respectively. (**C**) Surface expression of CD11b, CD15, CD16 and CD66b by differentiating cord blood- or bone marrow-derived cells (gated on live granulocytes as in A) in the presence (red) or absence (blue) of GM-CSF using flow cytometry. Representative histograms, and gray is isotype control (n = 3).

**Figure 4:**
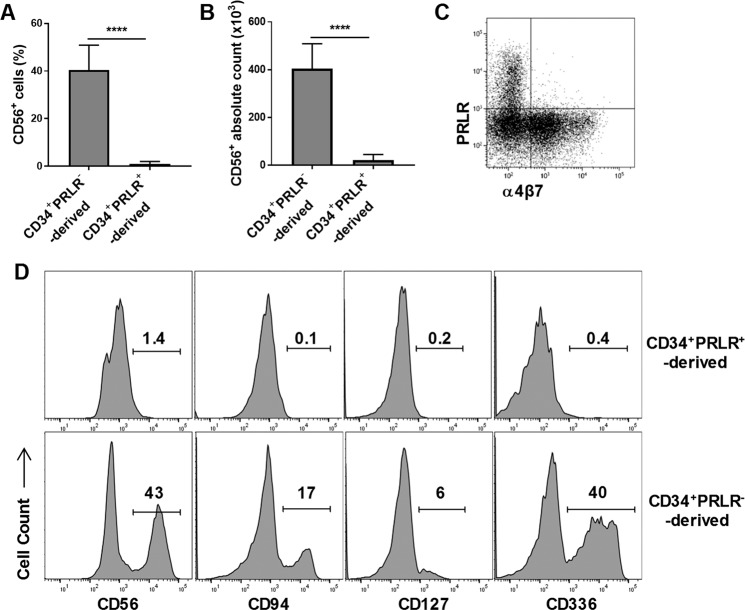
CD56^+^ lymphocytes generate from CD34^+^PRLR^−^ progenitors not CD34^+^PRLR^+^ cells. UCB-derived CD34^+^ HSCs were expanded for four days using FLT3L, LDL, SCF and TPO followed by FACS sorting into CD34^+^PRLR^+^ and CD34^+^PRLR^−^ subsets and differentiated for up to 28 days. (**A,B**) Cells were stained for ILCs surface markers at day 28 of culture and the percentage (**A**) and absolute number (**B**) of CD56^+^ cells are shown in bar graphs (n = 6). (**C**) Flow cytometry showing surface expression of integrin α4β7 by day 7 differentiating CD34^+^PRLR^+^ cells. Representative dot plots (n = 5). (**D**) Surface expression of CD56, CD94, CD127 and CD336 by differentiating CD34^+^PRLR^+^ cells (upper row) compared to CD34^+^PRLR^−^ cells (lower row) at day 28. Representative histograms and values represent the percentage (n = 6). (**A,B**) Data are shown as means ± SD, paired t-tests and significance is shown (**** = p < 0.0001).

To investigate the developmental origin of the CD34^+^PRLR^+^ cells, freshly isolated CD34^+^ HSCs were sorted into MLP, CMP, GMP and NK/BP (Fig. 2A)^55^, and cultured as described above. PRLR-expressing cells were mainly generated from the GMP compartment (Fig. 2B–D). At day 4 of culture, FACS sorted PRLR^+^ progenitors lacked all lineage markers except for CD15 and CD43, further supporting their myeloid origin (Fig. 2E)^56^. Evaluation of these cells at later time points (day + 14) showed that they remain CD15^+^, and acquired CD11a, CD11b, CD11c, CD16, CD18, CD45RO and CD123 (Fig. 2F). To study their differentiation, we sorted CD34^+^PRLR^+^ cells from four-day expanded CD34^+^ HSCs (as shown in Fig. 1A). As above, in the presence of ILC promoting cytokines (IL-3, IL-7, IL-15, IL-23, SCF and FLT3L) the CD34^+^PRLR^+^ progenitors differentiate into immature myeloid cells (Fig. 3A–C). Addition of GM-CSF at later times (day + 21) resulted in the generation of mature granulocytes expressing CD15, CD16 and CD66b (Fig. 3A–C). Previously, we showed that some myeloid progenitors could give rise to CD56^+^ lymphocytes^23^. However, sorted CD34^+^PRLR^−^, but not CD34^+^PRLR^+^ cells gave rise to CD56^+^ lymphocytes (Fig. 4A–C). Moreover, the CD34^+^PRLR^+^ progenitors do not express integrin α4β7, a marker that defines innate lymphoid precursors with the potential to develop into ILCs^12,15,16,57^ (Fig. 4C). In further support of these findings, the CD34^+^PRLR^+^ progenitors do not differentiate into CD94^+^, CD127^+^ and CD336^+^ lymphocytes (Fig. 4D). Collectively, these findings show that CD34^+^PRLR^+^ progenitors develop from the GMPs under the influence of SCF and FLT3L, thereby acquire various myeloid markers and seem to lack lymphoid potential.

### CD34^+^PRLR^+^ myeloid progenitors support CD56^+^ cells development from lymphoid progenitors

**Figure 5:**
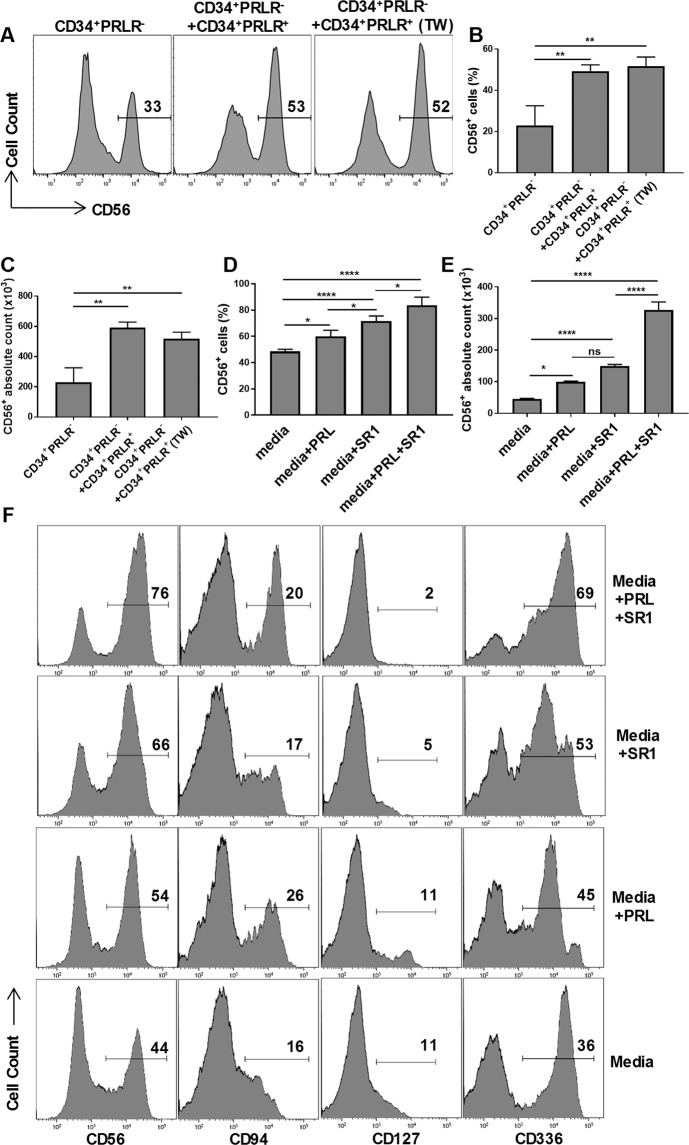
CD34^+^PRLR^+^ progenitors enhance the generation of CD56^+^ lymphocytes from CD34^+^PRLR^−^ progenitors. UCB-derived CD34^+^ HSCs were expanded for four days using FLT3L, LDL, SCF and TPO. Cells were then differentiated with or without PRL and SR1 for 21 days. The CD34^+^PRLR^+^ and CD34^+^PRLR^−^ cells were sorted at day 4 by FACS followed by differentiation for 21 days. CD34^+^PRLR^−^ and CD34^+^PRLR^+^ co-culture was done in a 2:1 ratios. (**A–C**) CD56 staining by CD34^+^PRLR^−^, CD34^+^PRLR^−^ + CD34^+^PRLR^+^ co-culture and CD34^+^PRLR^−^ + CD34^+^PRLR^+^ co-culture in transwell system at day 21. Representative histograms (**A**) percentage in bar graphs (**B**) and absolute number in bar graphs (**C**) of CD56^+^ cells are shown (n = 3). (**D,E**) Cells differentiating in the presence or absence of PRL and SR1 were stained for CD56 at day 21 of culture and the percentage (**D**) and absolute number (**E**) of CD56^+^ cells are shown in bar graphs (n = 7). (**F**) Surface expression of CD56, CD94, CD127 and CD336 by cells differentiating in the presence or absence of PRL and SR1 at day 21. Representative histograms and values represent the percentage (n = 7). (**B–E**) Data are shown as means ± SD, One-way ANOVA and significance is shown (* = p < 0.01; ** = p < 0.001; **** = p < 0.0001).

As above, CD34^+^PRLR^+^ myeloid progenitors differentiate into granulocytes, while CD56, CD94, CD127 and CD336 lymphocytes develop exclusively from the CD34^+^PRLR^−^ compartment of expanded HSCs (Fig. 4). To investigate whether the CD34^+^PRLR^+^ myeloid progenitors either positively or negatively influence CD34^+^PRLR^−^ differentiation, we separated these two populations early in the culture (day + 4) and performed co-culture experiments. Co-culture of CD34^+^PRLR^+^ cells with CD34^+^PRLR^−^ cells separated by a transwell membrane enhanced the generation of the CD56^+^ lymphocytes from the latter population in a contact independent manner (Fig. 5A–C). Moreover, addition of PRL into the CD34^+^ HSCs culture (which consists both CD34^+^PRLR^+^ and CD34^+^PRLR^−^ cells) further enhanced CD56^+^ lymphocyte differentiation (Fig. 5D–F). Interestingly, co-culture of bone marrow-derived CD34^+^PRLR^+^ cells with CD34^+^PRLR^−^ cells also enhanced the generation of the CD56^+^ lymphocytes, while addition of PRL further increases CD56^+^ lymphocyte differentiation (Supplementary Fig. 3A,B). Previously, stemregenin1 (SR1), an inhibitor of aryl hydrocarbon receptor (AHR), was demonstrated to enhance the generation of CD56^+^ lymphocytes from HSCs^58,59^. Consistent with these studies, SR1 increased the CD56^+^ cell differentiation from HSCs (Fig. 5D–F). Interestingly, addition of PRL along with SR1 into HSCs culture showed a synergistic activity on the generation of CD56^+^ cells (Fig. 5E). Finally, we show that PRL enhanced the differentiation of CD94 and CD336 expressing lymphocytes (Fig. 5F).

### PRL activates CD34^+^PRLR^+^ progenitors

**Figure 6:**
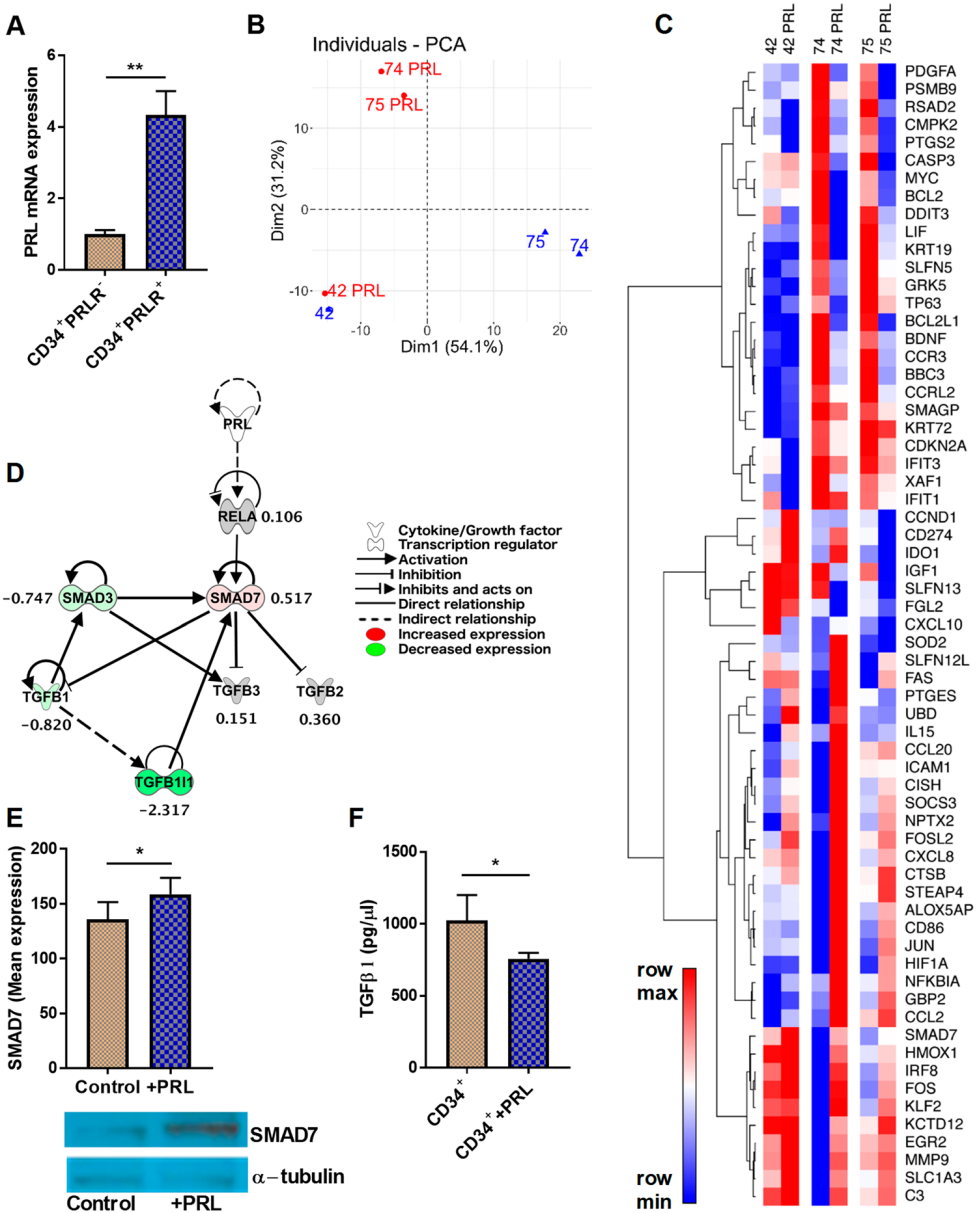
Activation of the CD34^+^PRLR^+^ progenitors by prolactin. UCB-derived CD34^+^ HSCs were expanded for 9 days using Flt3L, LDL, SCF and TPO, and CD34^+^PRLR^+^ cells were sorted using FACS. (**A**) Quantitative PCR and expression of PRL mRNA in CD34^+^PRLR^+^ cells is shown relative to its expression in CD34^+^PRLR^−^ cells after normalizing to the expression of GAPDH (n = 4/group). (**B**) RNAseq, principal component analysis of global gene expression and PCA plot is shown (n = 3). Prolactin treated (Red) and non-treated (Blue) were shown for donors 42, 74 and 75. (**C**) Heat map showing relative expression of PRL-regulated downstream genes found in the IPA causal network list (n = 3/group). (**D**) IPA generated network highlighting the relationship between PRL, SMAD7, and TGFβ-1. Values represent the log2 fold change between PRL treated and untreated samples (n = 3/group). (**E**) Sorted 9 days old CD34^+^PRLR^+^ cells were treated with prolactin or a control, PBS, for 48 hours and expression level of SMAD7 in control or treated cells was analyzed using western blot. Bar graph shows quantification of SMAD7 levels and mean expression in each condition. One representative western blot showing SMAD7 and the loading control, α-tubulin (n = 5). Both SMAD7 and α-tubulin were probed from the same gel/membrane. (**F**) ELISA and the concentration of TGFβ-1 in the supernatant of prolactin treated or control CD34^+^ culture is shown in bar graph (n = 4/group). (**A,E,F**) Data is shown as means ± SD, paired t-tests and significance is depicted (* = p < 0.05, ** = p < 0.01).

As shown in Fig. 1, CD34^+^PRLR^+^ progenitors specifically express PRLR both at the mRNA and protein level. Additionally, comparing to CD34^+^PRLR^−^ progenitors, CD34^+^PRLR^+^ progenitors expressed more PRL mRNA (Fig. 6A), perhaps suggesting autocrine activity of this molecule in CD34^+^PRLR^+^ progenitors^26,27,44^. These and the above findings prompted us to investigate the transcriptional influence of PRL on the CD34^+^PRLR^+^ myeloid cells by adding human recombinant PRL into the cultures. Sorted CD34^+^PRLR^+^ progenitors from 9-day expanded CD34^+^ HSCs (as shown in Fig. 1A) were stimulated with PRL for 48 hours and RNA-sequencing was performed. The principal component analysis (PCA) demonstrates that donors exhibited different responses to PRL treatment (Fig. 6B). Ingenuity pathway analysis (IPA) highlighted changes in PRL-associated transcriptional regulators such as IRF8, JUN, MYC, NFKBIA, RELA and TP63 (Fig. 6C,D). It also appeared that inflammation associated genes such as IL15 were among the PRL-regulated downstream genes in the causal network analysis (Fig. 6C)^60^. Interestingly, RNAseq data showed evidence of SMAD7 activation (Fig. 6C,D) and TGF-β1 inhibition (Fig. 6D) in PRL treated CD34^+^PRLR^+^ myeloid cells. Similarly, quantitative PCR showed that PRL treatment in CD34^+^PRLR^+^ myeloid cells derived from cord blood or bone marrow increases and decreases the expression of SMAD7 and TGF-β1, respectively (Supplementary Fig. 3C–D). Western blot confirmed increased production of SMAD7 protein by PRL stimulated CD34^+^PRLR^+^ cells (Fig. 6E). Also corroborating the RNAseq and quantitative PCR data, PRL stimulation inhibited the production of TGF-β1 from CD34^+^PRLR^+^ progenitors as shown by ELISA (Fig. 6F).

## Discussion

Human CD56^+^ lymphocytes differentiate from a common ILC progenitor that are downstream of multi-lymphoid progenitors^13,14,61^. Hematopoietic development necessitates distinct soluble factors that influence cell fate by acting on the progenitors at different stages of development^1–3,62^. The role of soluble molecules including IL-1β, IL-2, IL-3, IL-7, IL-15, IL-23, SCF and FLT3L during CD56^+^ lymphocyte development have been reported^9–14,61^. These cytokines are released from mature cells of both hematopoietic and non-hematopoietic lineages (including endothelium, bone marrow and lymphoid tissues)^4–6^. In addition to the well-established roles of cytokines and chemokines in lymphoid differentiation, the function of hormones, such as PRL, during hematopoiesis has also been reported^24–26,28^.

Both *in vivo* and *in vitro* studies show that PRL enhances the activation and maturation of lymphoid lineage cells^27,30,33,63–68^. However, *prlr*^*−/−*^ mice show appropriate proportions and numbers of all hematopoietic lineages indicating that, while this hormone may influence hematopoiesis, it is not necessary for lineage specification or differentiation^40,69^. Similarly, we find that while PRL is not needed for CD56^+^ lineage development, its addition increased the proportion and numbers of these cells in the culture. Because the PRLR signaling complex uses kinases and STATs it has been grouped among the type 1 cytokine receptor superfamily^36,38–40^. Apart from the mammary gland, immune cells also express this receptor^27,34,39,42,43^. PRL is mainly released by the anterior pituitary gland, however myeloid cells are non-endocrine sources of this hormone^27,34,35,68^. Interestingly, most myeloid cells co-express PRL and the PRLR, indicating the potential for both autocrine and paracrine actions^26,27,34,44^. Here, we also found that CD34^+^PRLR^+^ myeloid progenitors express PRL.

The expression of PRLR in a subset of tonsillar-derived human CD34^+^ HSC progenitors^24–26^ perhaps highlights the relevance of our findings and the importance of PRL during hematopoiesis, especially considering prior studies showing that secondary lymphoid tissues, such as the tonsil, are sites of CD56^+^ lineage development^70^. Additionally, murine studies show that PRL directly promotes hematopoiesis and accelerates immune reconstitution after BMT^24,28^. Studies also suggest an indirect role of PRL during lymphoid cell development, but the details remain unclear^28^. In this study, we show that SCF and FLT3L induce PRLR on CD34^+^-derived myeloid progenitors that initially lack PRLR expression. These cytokines (SCF and FLT3L) are well established to promote myeloid cell development^71,72^. Interestingly, CD34^+^PRLR^+^ progenitors also co-express PRL, supporting the possibility of autocrine action of PRL on these cells^34,44^. Despite this, exogenous recombinant PRL stimulated CD34^+^PRLR^+^ myeloid progenitors to augment CD56^+^ lymphocyte development. Despite the presence of ILC instructive cytokines (IL-7, IL-15, IL-23, SCF and FLT3L)^10,11,61^, CD34^+^PRLR^+^ progenitors differentiate into immature granulocytes (myelocytes), suggesting their loss of lymphoid potential. In further support of this, when GM-CSF is added, the cells develop into mature granulocytes. Exogenously added PRL activated the CD34^+^PRLR^+^ myeloid progenitors, resulting in IL-15 activation and a reduction in TGF-β1, possibly through SMAD7 activation^60^. These findings are in line with other data showing activation induced pro-inflammatory cytokine production in myelocytes^73,74^. We identified TGF-β1 as a soluble mediator derived from these CD34^+^PRLR^+^ myeloid progenitors, and its reduction during PRL treatment enhanced CD56^+^ lineage development from the committed lymphoid progenitors. In line with these findings, results from transwell co-culture experiments also strongly support a CD34^+^PRLR^+^ myeloid progenitor-derived soluble mediator that modulates CD56^+^ lymphocyte development from CD34^+^PRLR^−^ lymphoid progenitors. Thus, PRL acts on this intermediary myeloid cell population to significantly increase CD56^+^ lineage differentiation through the production of SMAD7, leading to the reduction in TGF-β1 and its signaling.

In summary, our findings show that PRL influences how HSCs from various sources (UCB, BM and PBSC) differentiate into the CD56^+^ lineage by acting on the CD34^+^PRLR^+^ myeloid progenitor in turn which produces SMAD7 and reduces the production of TGF-β1. We further show that exogenous PRL enhances the proportion and absolute numbers of CD56^+^ cells derived from CD34^+^ cells. Our findings are in line with previous human and murine studies, which implicate PRL as influencing the generation of lymphocytes, including CD56^+^ NK cells^25,26,28^. Herein, we demonstrate the indirect role of PRL in enhancing CD56^+^ lineage differentiation from CD34^+^ cells by favoring pro-inflammatory myeloid progenitors that express SMAD7 and reduce TGF-β1, known to impair NK cell differentiation^50–53^. These studies uncover how a heretofore unknown interaction between differentiating myeloid cells and NK cell precursors. They also provide data supporting a fundamental role for PRL in the development of NK cells, which has translational value considering that various groups are contemplating the use of induced pluripotent stem cell (iPSC)-derived, off the shelf NK cells for therapeutic purposes and hence the addition of PRL might augment yields of stem cell-derived NK cells.

## Methods

### Isolation and expansion of CD34^+^ HSCs

De-identified umbilical cord blood units were purchased from St. Louis Cord Blood Bank, while bone marrow and peripheral blood from male and female individuals (age range 8–24) were obtained from the cell bank in Colorado children’s hospital under institutional review board (IRB)-approved protocols. Mononuclear cells were isolated by density gradient centrifugation using Lymphoprep (Stemcell). The CD34^+^ HSCs were positively enriched using MACS CD34^+^ enrichment kit (Milteny). The cells (purity,>95%) were suspended (5 × 10^4^ cells/ml) in Stemspan serum free expansion medium II cell culture media (Stemcell) supplemented with 1% penicillin + streptomycin, SCF (100 ng/ml, R&D Systems), FLT3L (100 ng/ml, Stemcell), TPO (50 ng/ml, R&D Systems) and LDL (10ug/ml, Stemcell) and expanded in a 24 well plate for 4 days. After 4 days of culture the cells expanded ~2-fold in average while the proportion of CD34^+^ cells remained>95%.

### PRL treatment and differentiation of CD34^+^ HSCs

After 4 days of expansion, the entire population was considered for further differentiation. In additional experiments, expanded CD34^+^ HSCs were FACS sorted based on PRLR expression into CD34^+^PRLR^-^ and CD34^+^PRLR^+^ populations. Expanded CD34^+^ HSCs, sorted CD34^+^PRLR^−^ or sorted CD34^+^PRLR^+^ cells were cultured in a previously described B0 differentiation media^11^ supplemented with SCF (20 ng/ml, R&D Systems), IL-3 (5 ng/ml, Stemcell) only for the first week, IL-7 (20 ng/ml, R&D Systems), IL-15 (10 ng/ml, NIH), IL-23 (10 ng/ml, R&D Systems) and FLT3L (10 ng/ml, Stemcell). PRL (1 ng/ml, Stemcell) was used to assess the effect of this hormone on CD56^+^ lineage differentiation, and SR-1 (1 µM, Cellagen Technologies) was used as a positive control to stimulate the generation of CD56^+^ lymphocyte development^58,59^. Granulocyte-monocyte colony-stimulating factor (GM-CSF, 1 ng/ml, Shenandoah Biotechnology) was used to differentiate granulocytes. Cells (1 × 10^3^ cells/per well) were cultured in a 96 well u-bottom plate. For the co-culture experiments, sorted CD34^+^PRLR^−^ and CD34^+^PRLR^+^ cells (in 2:1 ratio) were co-cultured in contact-dependent or with a trans-well system using flat-bottom 96 well plates. Cells were cultured for a total of up to 28 days of differentiation.

### mRNA-sequencing

Umbilical cord blood (UCB)-derived CD34^+^ HSCs were expanded for 9 days, CD34^+^PRLR^+^ progenitors were FACS sorted followed by treatment with 1 ng/ml of PRL for 48 hours and RNA was extracted. Non-treated CD34^+^PRLR^+^ progenitors were used as controls. RNA from 1 × 10^6^ CD34^+^PRLR^+^ progenitors, was extracted using the RNeasy Mini Kit (Qiagen) according to the manufacturer specifications. RNA purity and concentration were measured on a NanoDrop (Thermofisher Scientific). Preparation of libraries, sequencing, alignment of reads and derivation of expression (FPKM) were as described before^16^. R v3.5.3 was used to analyze differential expression between PRL treated and control samples. Non-protein coding genes were identified by their absence from a protein coding gene list obtained from the Ensembl BioMart and were subsequently filtered from the dataset, along with genes that had no expression across all samples. A PCA plot was generated from all genes following filtration and a log10 transformation using the prcomp function in R. Log2 fold change was then calculated between PRL treated and control samples and genes with an absolute log2 fold change greater than 0.5 were used for pathway analysis using Ingenuity Pathway Analysis (IPA, Qiagen). A heatmap was produced, using Morpheus (Broad Institute), for the resulting target molecules found in the PRL causal network generated from the pathway analysis.

### Quantitative PCR (qPCR)

Cord blood, bone marrow or peripheral blood CD34^+^ HSCs were expanded for 9 days, CD34^+^PRLR^+^ progenitors were FACS sorted followed by treatment with 1 ng/ml of PRL for 48 hours and RNA was extracted. SMAD7 and TGF-β1 mRNA expression in the non-treated CD34^+^PRLR^+^ or PRL-treated CD34^+^PRLR^+^ progenitors were analyzed using qPCR. qPCR was also used to analyze PRL and PRLR mRNA expression in the CD34^+^ HSCs, sorted CD34^+^PRLR^+^ progenitors as well as sorted CD34^+^PRLR^−^ progenitors. Taqman gene expression assays for PRL (Hs00168730_m1), PRLR (Hs01061477_m1), SMAD7 (Hs00998193_m1) and TGF-β1 (Hs00998133_m1) were purchased from Thermofisher Scientific. The qPCR experiments were done as previously described^16^.

### Microscopic staining of cells

For Wright staining, 1 × 10^5^ differentiated CD34^+^PRLR^+^ cells were washed using cold 2% FCS-PBS twice and suspended in 100 μl of cold 1% BSA-PBS. Samples (100 μl each) were added into the cytospin wells, slides and filters were placed in the cytospin with the cardboard filters facing the center of the cytospin and centrifuged at maximum speed for 5 minutes. The filters were removed from the slides, dried and methanol was used to fix the smear, followed by Wright staining. The slides were examined using light microscope.

### Flow cytometry analysis and Western blot

Differentiation of myeloid cells and lymphoid cells were analyzed using flow cytometry. Flow cytometry-based viability assessment was performed using the fixable viability dye eFluor 780 (eBioscience). Expression of surface receptors was determined using the following monoclonal antibodies: anti-CD3-PerCp5.5 (clone HIT3a), anti-CD10-FITC (clone HI10a), anti-CD11c-PerCp5.5 (clone Bu15), anti-CD15- APC/BV605 (clone W6D3), anti-CD16-FITC (clone 3G8), anti-CD19-PerCp5.5 (clone HIB19), anti-CD38-PE (clone HIT2), anti-CD43-PE (clone CD43–10G7), anti-CD66b-PECy7 (clone G10F5), anti-CD127-PE (clone A019D5), anti-CD336-APC (clone P44–8) and anti-mouse IgG-FITC/PE (clone Poly4053) (all from Biolegend); anti-CD11a-FITC (clone G43–25B), anti-CD11b-FITC/PE (clone M1/70), anti-CD18-APC (clone 6.7), anti-CD34-PE/APC (clone 4H11), anti-CD45-APC/PE (clone HI30), anti-CD45RO-PerCp5.5 (clone UCHL1), anti-CD56-BV421/BV605 (clone NCAM16.2), anti-CD94-PerCp5.5/FITC (clone HP-3D9) and anti-CD123-FITC (clone 7G3) (all from BD Biosciences); anti-PRLR-PE/APC (clone B6.2 + PRLR742), from Novus Biosciences); anti-CD45RA-PerCp5.5 (clone HI100, from Tonbo Biosciences); and anti-α4β7 (Cat# 11718, from NIH AIDS reagent program). Isotype-matched antibodies from the respective companies were used as negative controls. Cell acquisition was performed in LSR II and data were analyzed using Flowjo (BD Biosciences) or Kaluza (Beckman Coulter) analysis software. Western blotting was used to assess the level of SMAD7 protein in PRL treated CD34^+^PRLR^+^ cells compared to that of untreated cells. Accordingly, sorted 9 day old CD34^+^PRLR^+^ cells were treated with PRL for 48 hours followed by washing with PBS and lysing using RIPA cell lysis buffer (Thermofisher Scientific) supplemented with protease inhibitors (Roche Diagnostics). After quantification, 45 µg of protein lysate was loaded per well in a Bis-Tris Gel (Invitrogen) to run for 1 hour at 120 V, transferred to a nitrocellulose membrane (Invitrogen) and then probed for SMAD7. Following exposure, the membrane was stripped using western blot Stripping Buffer (ThermoFisher) and washed with Tris-buffered saline containing 0.1% Tween 20 prior to being probed for the α-tubulin loading control. SMAD7 and α-tubulin antibodies were purchased from R&D Systems and Cell signaling, respectively.

## Supplementary information

Supplementary information.
