# Supplementary material for: Prolactin Acts on Myeloid Progenitors to Modulate SMAD7 Expression and Enhance Hematopoietic Stem Cell Differentiation into the NK Cell Lineage
Source: Sci Rep. 2020 Apr 14;10:6335. doi: 10.1038/s41598-020-63346-4 (PMC7156717; doi:10.1038/s41598-020-63346-4)
Supplement: Supplementary file 1 — Supplementary information. [file 41598_2020_63346_MOESM1_ESM.pdf]

Prolactin Acts on Myeloid Progenitors to Modulate SMAD7 Expression and Enhance Hematopoietic Stem Cell Differentiation into the NK Cell Lineage

Dejene M. Tufa, Tyler Shank, Ashley M. Yingst, George Devon Trahan, Seonhui Shim, Jessica Lake, Renee Woods, Kenneth Jones and Michael R. Verneris*

Affiliations:

All authors are members of the University of Colorado and Children's Hospital of Colorado, Department of Pediatrics, Center for Cancer and Blood Disorders. Research Complex 1, North Tower, 12800 E. 19th Ave., Mail Stop 8302, Room P18-4108, Aurora, CO 80045

*Corresponding Author:

Michael R. Verneris, MD
Pediatric Bone Marrow Transplantation and Cellular Therapy
University of Colorado Anschutz Medical Campus
Research Complex 1, North Tower
12800 E. 19th Ave.
Mail Stop 8302
Room P18-4108
Aurora, CO 80045
E-mail: Michael.Verneris@CUAnschutz.edu

Supplementary Information

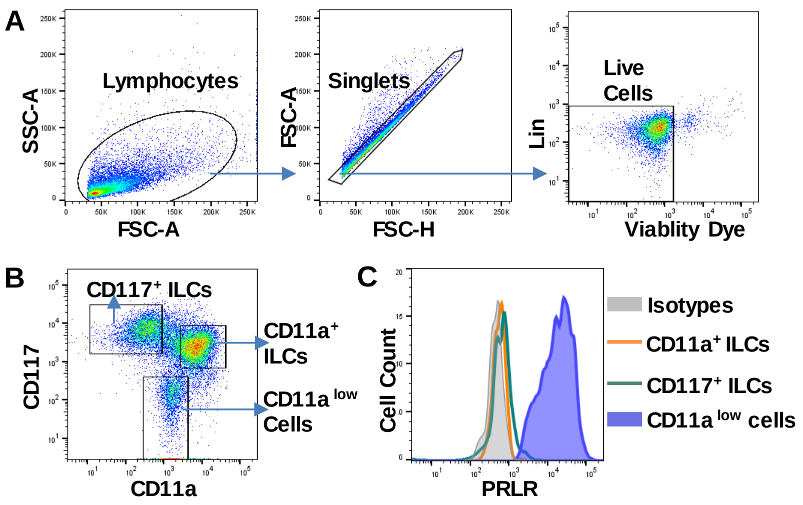

Supplementary Figure 1. PRLR expression by differentiating HSCs. HSCs were differentiated for 21 days in the influence of cytokine cocktails that lead to ILCs differentiation (IL-3, IL-7, IL-15, IL-23, SCF and Flt3L). (**A and B**) Cells were stained for CD11a and CD117, and the CD117^{+} ILCs, CD11a^{+} ILCs and CD11a^{low} cells is shown in representative dot plots. (**C**) PRLR expression of CD117^{+} ILCs, CD11a^{+} ILCs and CD11a^{low} cells is shown in representative histograms.

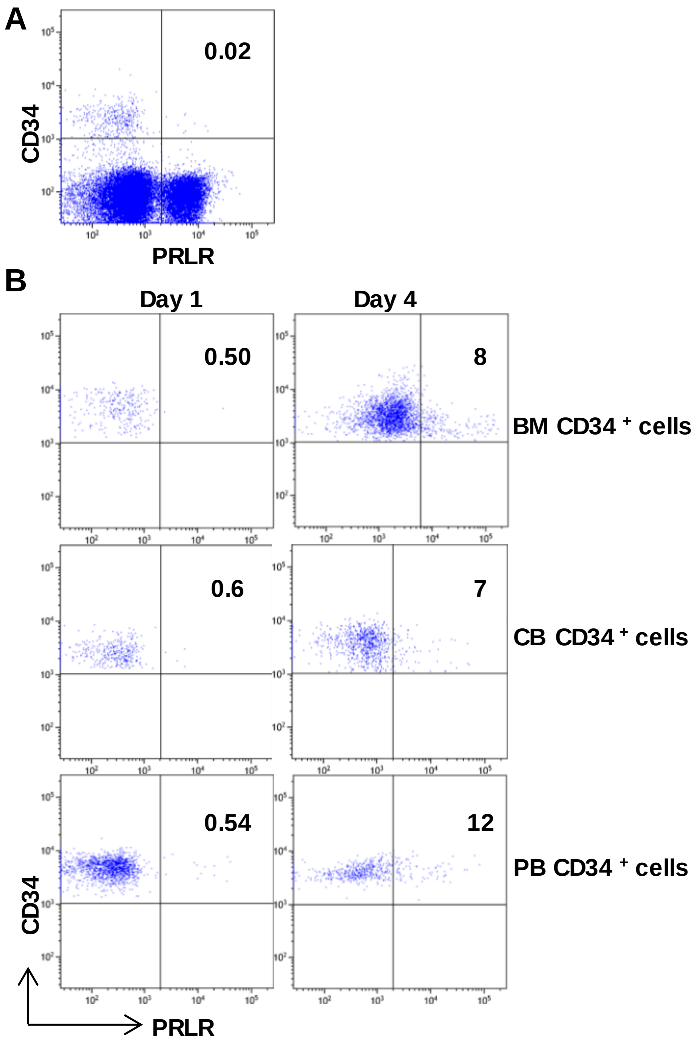

Supplementary Figure 2. Acquisition of PRLR by HSCs of different sources. Freshly isolated HSCs were differentiated for 4 days in the influence of cytokine cocktails that lead to HSCs expansion (SCF, TPO, LDL and FLT3L). (**A**) Freshly isolated cord blood peripheral mononuclear cells were stained for CD34 and PRLR, and representative dot plots is shown. (**B**) Bone marrow (BM), cord blood (CB) and peripheral blood HSCs were stained for CD34 and PRLR, and representative dot plots is shown for day 1 and day 4. Values show percentage of $CD34^{+}PRLR^{+}$ cells.

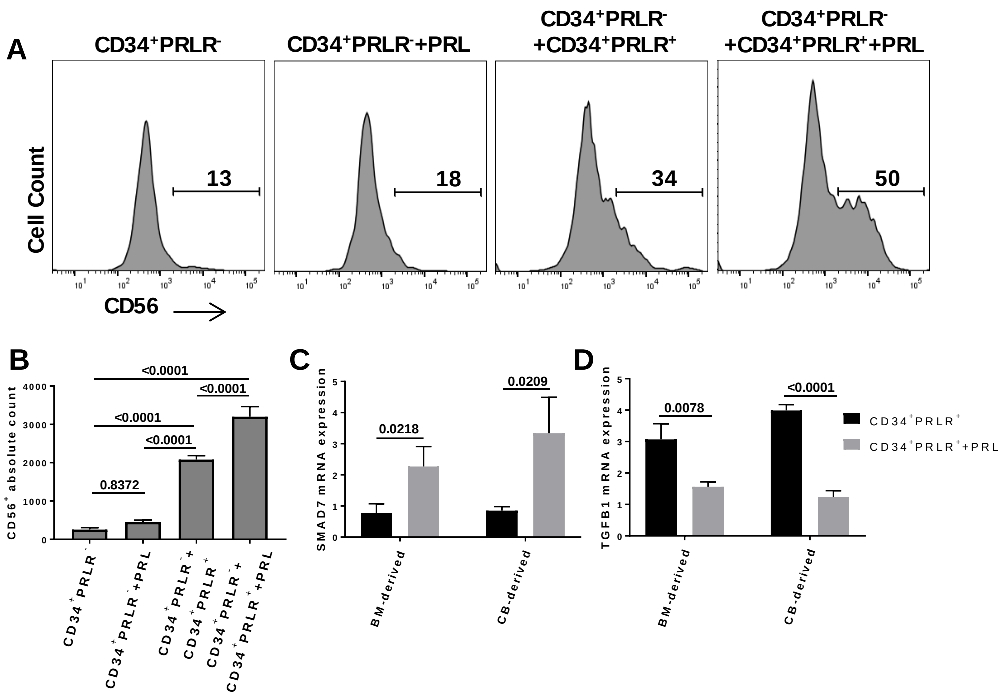

Supplementary Figure 3. PRL influences CD34$^+$PRLR$^+$ progenitors to enhance the generation of CD56$^+$ lymphocytes. Bone marrow and cord blood CD34$^+$ HSCs were expanded for 9 days using FLT3L, LDL, SCF and TPO. The CD34$^+$PRLR$^+$ and CD34$^+$PRLR$^-$ cells were sorted by FACS followed by differentiation for 21 days with or without PRL. CD34$^+$PRLR$^-$ and CD34$^+$PRLR$^+$ co-culture was done in a 2:1 ratios. (**A**) Representative histograms (n=3) for CD56 staining of bone marrow-derived differentiating CD34$^+$PRLR$^-$ or CD34$^+$PRLR$^-$+CD34$^+$PRLR$^+$ co-culture at day 21. Values represent percentage of CD56$^+$ cells. (**B**) Absolute number of CD56$^+$ cells in bar graphs (n=3/group). (**C and D**) Day 9 sorted bone marrow- or cord blood-derived CD34$^+$PRLR$^+$ cells were either treated or untreated with PRL for 48 hrs. Quantitative PCR and expression of SMAD7 (**C**) or TGFB1 (**D**) mRNA in PRL treated CD34$^+$PRLR$^+$ cells is shown relative to its expression in untreated CD34$^+$PRLR$^+$ cells after normalizing to the expression of GAPDH (n=3/group). (**B-D**) Data is shown as means ± SD, One-way ANOVA (**B**) or paired t-tests (**C** and **D**) and p-value is depicted.
